# StemRegenin-1 Attenuates Endothelial Progenitor Cell Senescence by Regulating the AhR Pathway-Mediated CYP1A1 and ROS Generation

**DOI:** 10.3390/cells12152005

**Published:** 2023-08-05

**Authors:** Hye Ji Lim, Woong Bi Jang, Vinoth Kumar Rethineswaran, Jaewoo Choi, Eun Ji Lee, Sangmi Park, Yeoreum Jeong, Jong Seong Ha, Jisoo Yun, Young Jin Choi, Young Joon Hong, Sang-Mo Kwon

**Affiliations:** 1 Laboratory for Vascular Medicine and Stem Cell Biology, Department of Physiology, Medical Research Institute, School of Medicine, Pusan National University, Yangsan 50612, Republic of Korea; dla9612@naver.com (H.J.L.); jangwoongbi@naver.com (W.B.J.); vinrebha@gmail.com (V.K.R.); wozh1304@naver.com (J.C.); easy2697@naver.com (E.J.L.); smpark7257@gmail.com (S.P.); suny1452@gmail.com (Y.J.); hajongseong@gmail.com (J.S.H.); jsyun14@hanmail.net (J.Y.); 2 Convergence Stem Cell Research Center, Pusan National University, Yangsan 50612, Republic of Korea; 3 Department of Hemato-Oncology, School of Medicine, Pusan National University, Yangsan 50612, Republic of Korea; chyj@pusan.ac.kr; 4 Department of Cardiology, Chonnam National University School of Medicine, Chonnam National University Hospital, Gwangju 61469, Republic of Korea

**Keywords:** StemRegenin-1, human endothelial progenitor cells, AhR pathway, reactive oxygen species, replicative senescence

## Abstract

Endothelial progenitor cell (EPC)-based stem cell therapy is a promising therapeutic strategy for vascular diseases. However, continuous in vitro expansion for clinical studies induces the loss of EPC functionality due to aging. In this study, we investigated the effects of StemRegenin-1 (SR-1), an antagonist of aryl hydrocarbon receptor (AhR), on replicative senescence in EPCs. We found that SR-1 maintained the expression of EPC surface markers, including stem cell markers, such as CD34, c-Kit, and CXCR4. Moreover, SR-1 long-term-treated EPCs preserved their characteristics. Subsequently, we demonstrated that SR-1 showed that aging phenotypes were reduced through senescence-associated phenotypes, such as β-galactosidase activity, SMP30, p21, p53, and senescence-associated secretory phenotype (SASP). SR-1 treatment also increased the proliferation, migration, and tube-forming capacity of senescent EPCs. SR-1 inhibited the AhR-mediated cytochrome P450 (CYP)1A1 expression, reactive-oxygen species (ROS) production, and DNA damage under oxidative stress conditions in EPCs. Furthermore, as a result of CYP1A1-induced ROS inhibition, it was found that accumulated intracellular ROS were decreased in senescent EPCs. Finally, an in vivo Matrigel plug assay demonstrated drastically enhanced blood vessel formation via SR-1-treated EPCs. In summary, our results suggest that SR-1 contributes to the protection of EPCs against cellular senescence.

## 1. Introduction

Endothelial progenitor cells (EPCs) have been proposed as promising resources for stem cell-based therapies because of their ability to differentiate into endothelial cells and contribute to neovascularization [1,2]. Studies have been conducted on EPCs for culture systems, surface markers, origin, and differentiation hierarchy [3]. Further studies have shown that EPCs are involved in regeneration and the healing of injured tissues [4,5]. In accordance with the trend of generation, attempts to apply existing studies, such as new markers and analysis techniques, are also being conducted [6,7]. Subsequent studies have suggested that EPCs are therapeutic agents for vascular disease. In various preclinical and clinical trials, cellular aging led to a decrease in cell yield, loss of function, and engraftment of cells after transplantation for stem cell therapy [8,9]. Various studies have been conducted to overcome the aging of EPCs [10,11,12,13], but it remains unclear. Cellular senescence, a state of irreversible growth arrest first described in vitro by Hayflick in 1961 [14], is caused by several factors, such as DNA damage, telomere shortening, oxidative stress, and related gene expression [15]. These risk factors are induced and accelerated by ROS [16]. In contrast, appropriate levels of ROS are offered as essential signaling molecules; however, excessive ROS-mediated damage to the cells has been implicated in neurodegeneration, atherosclerosis, aging, and other diseases [17]. Moreover, because of their location, endothelial cells are constantly exposed to flowing blood that contains various factors that can cause senescence, including oxygen, ROS, and inflammatory cytokines. These effects can induce cellular senescence [18]. Thus, overcoming endothelial cellular senescence is essential.

StemRegenin-1 (SR-1), an antagonist of the aryl hydrocarbon receptor (AhR), was identified in an unbiased screen for compounds with primary human hematopoietic stem cells. SR-1 has been shown to promotes the expansion of CD34+ cells through direct binding and inhibition of the AhR signaling pathway [19]. Moreover, a phase 1/2 trial was conducted, which resulted in the expansion of CD34+ cells, leading to rapid engraftment in the assessed patients [20]. In addition, recent studies on SR-1 in hematopoietic stem/progenitor cells and hematopoietic lineages have followed [21,22,23]. AhR is a transcription factor that regulates gene expression in an exo/endogenous ligand-dependent manner [24]. It has been verified as an important modulator at the cellular level, including cell differentiation, pluripotency, and stemness in normal or tumor cells [25]. AhR plays a crucial role in skin regeneration and homeostasis by controlling epidermal stem cells [26]. AhR ligands affect the maintenance of undifferentiated human embryonic and induced pluripotent stem cells [27]. It has been shown that AhR is an attractive target of breast cancer [28]. Moreover, AhR is associated with liver, fibroblast, and vascular aging [29,30,31]. In previous studies, AhR activation was induced by its binding to affinitive ligands, causing induction of the cytochrome P450 (CYP) enzyme, which is responsible for increasing ROS generation [32,33]. In addition, CYP genes related to the aging process and ROS formation have been described [34]. Based on these studies, SR-1 could contribute to the attenuation of EPC senescence by antagonizing AhR-mediated ROS generation. However, the potential protective effects of SR-1 on senescence of EPCs have not yet been described. In this study, we investigated the effects of SR-1 on replicative senescence in EPC. We demonstrated that SR-1 preserved EPC characteristics and functional ability in the face of long-term ex vivo expansion and oxidative stress. These findings provide insights into the treatment of vascular diseases.

## 2. Materials and Methods

### 2.1. Isolation and Culture of Human Endothelial Progenitor Cells

Human umbilical cord blood (HUCB) was provided by Pusan National University Yangsan Hospital (PNUYH, IRB No. 05-2017-053), and mononuclear cells (MNCs) were isolated from HUCB using density gradient centrifugation with Ficoll (GE Healthcare, Buckinghamshire, UK). The freshly isolated MNCs were seeded in dishes coated with 1% gelatin (Sigma-Aldrich, St. Louis, MO, USA) and cultured in endothelial growth medium-2 (EGM-2; Lonza, Walkersville, MD, USA) consisting of endothelial cell basal medium (EBM-2), 5% fetal bovine serum (FBS), 1% penicillin-streptomycin, human basic fibroblast growth factor (bFGF), human vascular endothelial growth factor, human insulin-like growth factor-1 (IGF-1), ascorbic acid, human epidermal growth factor (EGF), and gentamicin sulfate-amphotericin (GA-1000). Cells were incubated at 37 °C in 5% CO_2_. After 5 days, nonadherent cells were discarded, and the attached cells were cultured continuously. The cultures were maintained to allow the development of spindle-shaped colonies. EGM-2 was changed daily, and the colonies were replated and cultured further. To compare young and senescent EPCs, young EPCs were used at passages ≤ 8, and senescent EPCs were used at passages ≥ 14 in subsequent experiments. 6-Formylindolo 3 2-b carbazole (FICZ) was from Sigma-Aldrich.

### 2.2. SR-1 Treatment

SR-1 was purchased from Peprotech (Rocky Hill, NJ, USA). The SR-1 stock solution was diluted to the final concentration (1 μM) of EGM-2. SR-1 treatment typically started at passage 6 for the long-term treatment experiment. The medium with SR-1 was replaced every day. When the cells reach approximately 90% confluence, they are sub-cultured to a new plate. Senescent EPCs for long-term treatment experiments were used from passage 14.

### 2.3. Cell Viability Assay

Cells were seeded in 96-well plates at a density of 5 × 10^3^ cells/well and grown for 48 h in EGM-2 medium with different concentrations of SR-1. Cell viability was evaluated using the CCK cell viability assay kit (Dongin, Seoul, Republic of Korea), according to the manufacturer’s instructions. CCK solutions were added and incubated for 1 h, and the absorbance was measured at 450 nm using a microplate reader (TECAN, Mannedorf, Switzerland).

### 2.4. Flow Cytometry Analysis (FACS)

Cells were suspended in FACS buffer (2% FBS, 2 mM EDTA in PBS) and stained with antibodies against CD34, CXCR4, VEGFR2, VE-cadherin (BD Biosciences, Franklin Lakes, NJ, USA), and c-Kit (Miltenyi Biotec, Bergisch Gladbach, Germany). The cells were then incubated at 4 °C for 30 min in the dark. The cells were then washed and resuspended in the FACS buffer. The samples were analyzed using flow cytometry (Accuri C6, BD Biosciences).

### 2.5. EdU (5-Ethynyl-2′-Deoxyuridine) Cell Proliferation Assay

EPCs were cultured on coverslips in a 6-well plate containing 10 μM EdU for 5 h. EdU staining was conducted using the EdU Cell Proliferation Kit (Invitrogen, Carlsbad, CA, USA), according to the manufacturer’s protocol. The nuclei were stained with Hoechst-33342 (Sigma-Aldrich). An automated microscope (BioTek, Winooski, VT, USA) was used to acquire the images.

### 2.6. Senescence-Associated β-Galactosidase (SA β-Gal) Assay

Senescent EPCs were seeded at 1.5 × 10^5^ cells on a 6-well plate. The cells were stained using a SA β-gal staining kit (Cell Signaling Technology, Denvers, MA, USA), according to the manufacturer’s instructions. After staining, the images were acquired using an automated microscope (BioTek), and the cells were counted with blue-colored staining for senescence-associated β-galactosidase activity.

### 2.7. Western Blot Analysis

Cells were lysed in RIPA lysis buffer containing a protease inhibitor cocktail (Thermo Fisher Scientific, Rockford, IL, USA). Equal amounts of protein were separated using sodium dodecyl sulfate-polyacrylamide gel electrophoresis (SDS-PAGE) and transferred to polyvinylidene fluoride (PVDF) membranes (Millipore, Billerica, MA, USA). The membrane was blocked with 5% skim milk and incubated with primary antibodies against γH2A.X (Abcam, Cambridge, UK), SMP30, p53 (Cell Signaling Technology), p21, and β-actin (Santa Cruz Biotechnology, Dallas, TX, USA) overnight at 4 °C. Membranes were incubated with a horseradish peroxidase (HRP)-conjugated secondary antibody (Enzo Life Sciences, Farmingdale, NY, USA). Protein bands were visualized using a chemiluminescent HRP detection reagent (Millipore) and imaged using an Amersham Imager (GE Healthcare).

### 2.8. Quantitative Real-Time Polymerase Chain Reaction (qRT-PCR)

Total RNA was isolated using TRIzol (Invitrogen). The isolated RNA was converted to cDNA using the cDNA Synthesis Kit (Takara, Kusatsu, Japan). To quantify mRNA levels, qRT-PCR was performed on a QuantStudio 3 (Applied Biosystems, Waltham, MA, USA) using a Power SYBR Green PCR Master Mix (Applied Biosystems). All experiments were performed according to the manufacturer’s instructions. Relative gene expression was normalized to the expression of the housekeeping gene GAPDH. The primer sequences used are listed in Table S1.

### 2.9. Scratching Wound Healing Assay

EPCs were seeded in 6-well plates, grown until confluence, and scratched using a scratcher (SPL Life Science, Pocheon, Republic of Korea) in each well, after which the cells were washed with EGM-2 medium. The cell plates were maintained in EGM-2 medium and incubated at 37 °C. Images of the migrated cells were captured using a light microscope (Olympus, Tokyo, Japan).

### 2.10. Transwell Migration Assay

Transwell migration assays were performed using 24-well Transwell 8.0 μm pore polycarbonate membrane inserts (Corning, NY, USA). The upper inserts were seeded at 5 × 10^4^ cells in EGM-2 medium and lower chamber was added with EGM-2 with 100 ng/mL SDF-1α (R&D Systems, Minneapolis, MN, USA). After incubating the plates for 6 h at 37 °C, the cells were fixed in 4% paraformaldehyde (PFA) and stained with 0.5% crystal violet in 25% methanol. The inserts were washed with distilled water until clean, and the insert membranes were mounted on glass slides. The stained cells were observed using an automated microscope (BioTek).

### 2.11. Tube Formation Assay

Matrigel (Corning) was coated onto 96-well plates and incubated at 37 °C for 30 min. EPCs were seeded at 1 × 10^4^ cells/well and incubated at 37 °C. The lengths of the tube branches were visualized at one microscopic field per well using a light microscope (Olympus).

### 2.12. ROS Measurements

To measure ROS levels, cell-permeant, 2′,7′-dichlorodihydrofluorescein diacetate (H_2_DCFDA) (Thermo Fisher Scientific), was used. The detached cells were incubated with 1 μM H_2_DCFDA for 30 min at 37 °C. Subsequently, the cells were washed with PBS and analyzed using flow cytometry (Accuri C6; BD Biosciences). The cell plates were incubated with 10 μM DCFDA at 37 °C for 30 min. Hoechst 33342 was used for nuclear staining. After washing with PBS, images were analyzed using an automated microscope (BioTek).

### 2.13. Immunofluorescence

EPCs were fixed in 4% PFA for 15 min at room temperature. Cells were washed, permeabilized with 0.1% Triton X-100 in PBS for 20 min and washed with PBS. Additionally, the cells were blocked with 5% normal goat serum in PBS + 0.1% Tween 20 for 1 h at room temperature. γH2A.X (Abcam) antibody was diluted with 5% BSA in PBS and incubated overnight at 4 °C. The next day, the slides were washed with PBS and incubated with the secondary antibody, Alexa Fluor-555 (Invitrogen), at room temperature for 1 h in the dark. The washed slides were mounted with DAPI. The stained cells were observed using an automated microscope (BioTek).

### 2.14. Matrigel Plug Assay

Matrigel plug assay was performed on BALB/c nude mice (Orient Bio, Seongnam, Republic of Korea). Cells (5 × 10^5^ cells) were added to 500 μL of growth factor-reduced Matrigel (Corning) containing 50 unit/mL heparin (Sigma-Aldrich) and then, subcutaneously injected into mice. Six days later, the mice were anesthetized, and Matrigel plugs were obtained. Matrigels were fixed with 4% PFA and embedded in paraffin. For immunostaining, Matrigel sections were stained with CD31 antibody (Abcam). Sections were imaged using an automated microscope (BioTek).

### 2.15. Statistical Analysis

All data were expressed as mean ± standard deviation (SD). Statistical significance was analyzed using student’s *t*-test, and differences with *p* < 0.05 were considered statistically significant.

## 3. Results

### 3.1. Characterization of SR-1 Treatment on Human Endothelial Progenitor Cells

To confirm the effect of SR-1 treatment on EPC, we assessed cell viability and phenotype. We treated with different concentrations of SR-1 for 24 and 48 h and selected concentration of SR-1 that did not affect cell viability for progress of the study (Figure S1). In addition, we observed no changes in the cell morphology of the selected SR-1 concentration-treated EPCs compared to the controls (Figure S2). The expression of isolated EPC surface markers also did not differ between the SR-1 and control groups (Figure S3).

### 3.2. Maintenance of CD34 and Progenitor Population of SR-1 Treated Endothelial Progenitor Cells

**Figure 1:**
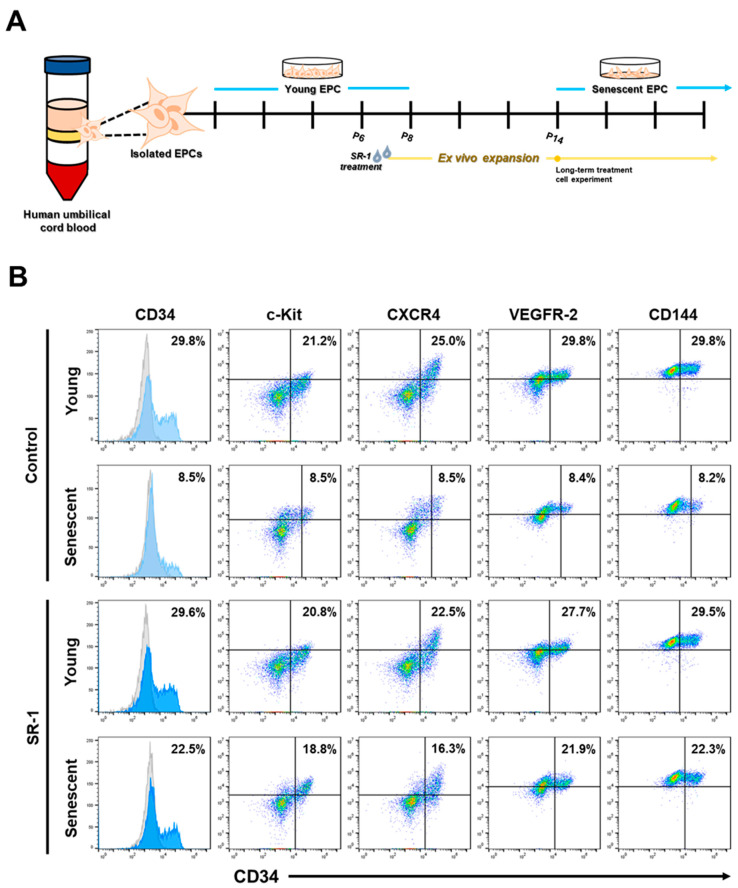
Effects of SR-1 long-term treatment on the expression of surface markers in EPCs. (**A**) Schematic representation to show isolation of EPCs from the human umbilical cord blood and ex vivo expansion with SR-1 treatment for experiment. (**B**) Expression of EPC surface marker (CD34, c-kit, CXCR4, VEGFR-2, CD144) was measured using flow cytometry analysis of young and senescent cells with continuous treatment of SR-1. (SR-1 1 μM treatment).

Together with the experiments in the supplementary figure, EPCs derived from the HUCB were investigated by treating with SR-1 during ex vivo expansion (Figure 1A). CD34 is a transmembrane phosphoglycoprotein that is frequently used as a marker for Hematopoietic stem and progenitor cells [35]. CD34 is a marker for many other cell types, including mesenchymal stromal cells, epithelial progenitors, and vascular endothelial progenitors. Thus, CD34 is recognized as a progenitor marker [36,37]. According to previous studies on CD34+ cell expansion with SR-1 [19], we first examined CD34+ cell populations during long-term culture with SR-1 from young passages at the initial stage to senescent passages. Interestingly, SR-1 maintained CD34+ cell populations, as well as in the initial stage, compared to the controls. The expression of other markers was preserved in SR-1-treated EPCs compared to that in the controls (Figure 1B).

### 3.3. Retaining Characteristics of SR-1-Treated EPCs

**Figure 2:**
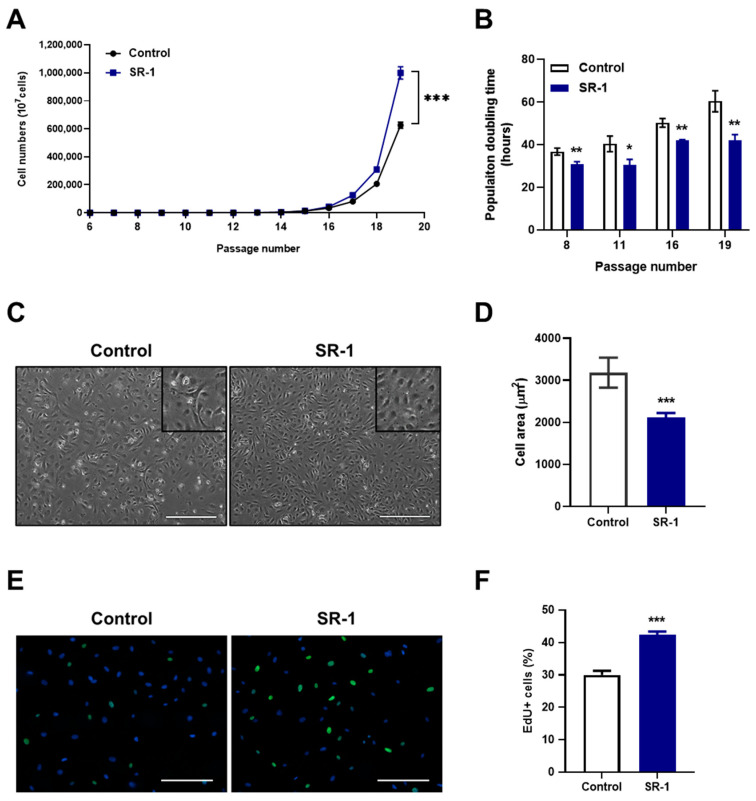
Effects of SR-1 long-term treatment on EPCs properties. (**A**) The graph demonstrates accumulative cell numbers of continuous SR-1 treatment. (**B**) Population doubling time of SR-1-continuously treated EPCs. (**C**) Representative phase-contrast images for long-term culture of EPCs with continuous SR-1 treatment (Scale bar, 400 μm). The boxed regions are shown at a high magnification (×2) in the inset. (**D**) Cell morphometric parameters measuring cell area. (**E**) Merged images of EdU staining (green) and Hoechst-33342 (blue). (**F**) Quantitative assessments of EdU-positive cells. The data are presented as mean ± standard deviation. (* *p* < 0.05, ** *p* < 0.01 and *** *p* < 0.001 vs. control, SR-1 1 μM treatment).

Along with the analysis of EPC surface markers, the yield of cells was significantly higher (Figure 2A), and the doubling time was maintained more steadily from young to senescent passages in SR-1 long-term-treated EPCs than in the controls (Figure 2B). In addition, the morphology of SR-1 long-term-treated EPCs was smaller in the cell area preserved in the existing cobble stone-like shape (Figure 2C,D). As expected, SR-1 long-term-treated EPCs showed improved proliferative capacity by confirming the increased EdU-positive cells compared to the controls (Figure 2E,F). Therefore, SR-1 not only maintains the CD34 population, which is recognized as a progenitor marker of EPCs, but also contributes to the maintenance of the EPC characteristics.

### 3.4. Attenuation of SR-1 Treatment on Replicative Senescence in EPCs

**Figure 3:**
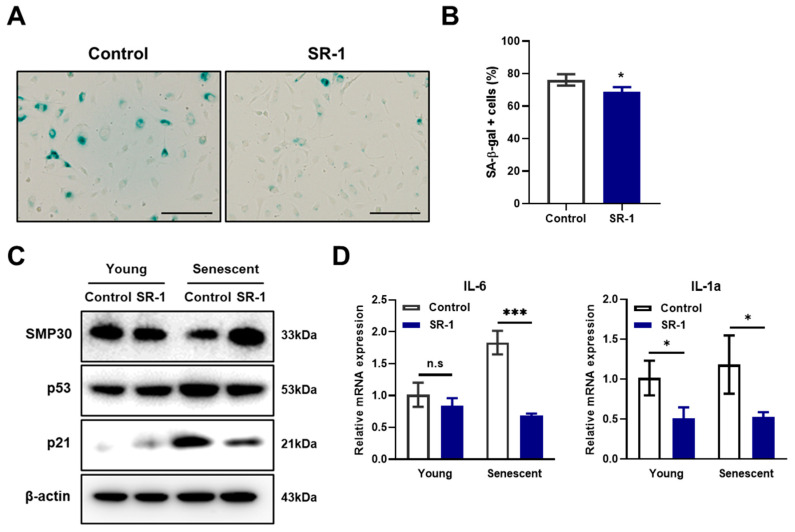
Effects of SR-1 on EPCs senescence. (**A**) SA-β-gal activity was measured in SR-1-continuously treated EPCs (Scale bar, 200 μm). (**B**) Percentage of SA-β-gal+ cells. (**C**) The expression levels of senescence-associated proteins were checked using western blot analysis in young and senescent EPCs. (**D**) Expression of SASP marker, Interleukin-6 and Interleukin-1α as examined by qRT-PCR in young and senescent EPCs. The mRNA levels were normalized to GAPDH. The data are presented as mean ± standard deviation. (n.s represents no significance, * *p* < 0.05 and *** *p* < 0.001 vs. control, SR-1 1 μM treatment).

A reduction in cell yield, cell area enlargement, and decrease in proliferation are representative aging phenotypes of cells [8]. It was confirmed that senescence associated β-galactosidase (SA-β-gal) activity, a characteristic of senescent cells, was decreased in SR-1 long-term-treated EPCs compared to the controls (Figure 3A,B). To determine whether SR-1 improves EPC senescence, we investigated the effect of SR-1 short-term treatment for 48 h on senescent EPCs. The expressions of the anti-senescence protein, SMP30, the pro-senescence protein, p21, and p53 were assessed at the protein level. Following treatment of EPCs with SR-1, SMP30 expression increased, whereas p21 and p53 expression decreased (Figure 3C). SR-1 treatment also decreased the mRNA expression of the SASP, which is a phenotype associated with senescent cells, including interleukin-6 (IL-6) and interleukin-1α (IL-1α) (Figure 3D). These data suggest that SR-1 attenuates cellular senescence in EPCs.

### 3.5. Enhancement of Migration and Tube-Forming Capacity in SR-1-Treated Senescent EPCs

**Figure 4:**
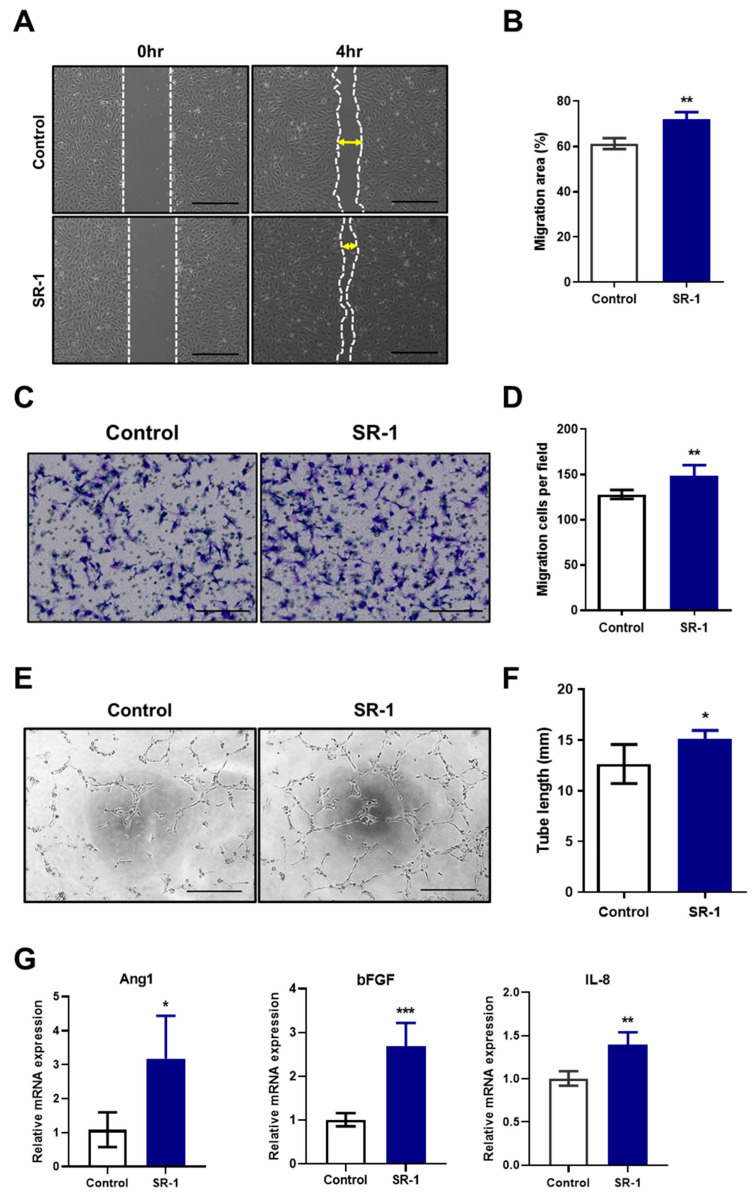
Effects of SR-1 on functional ability in senescent EPCs. (**A**) Representative image of the effect of SR-1 on migration of senescent EPCs. Cell migration was evaluated using the scratch-wound healing assay (scale bar, 400 μm). (**B**) Percentage of migration area is expressed. (**C**) Transwell migration assay was performed to evaluate the migration ability of senescent EPCs (scale bar: 200 μm). (**D**) Quantitative data of the number of migrated cells. (**E**) The tube-forming ability of senescent EPCs on Matrigel was assessed using a tube-formation assay (scale bar, 400 μm). (**F**) Quantification of the total tube length of tube-like structures. (**G**) Relative quantification of gene expression of the proangiogenic factors, Ang1, bFGF, and IL-8 in senescent EPCs. mRNA levels were normalized to those of GAPDH to assess the effects of SR-1 on EPCs senescence, EPCs were used at passages ≥ 12. Data are presented as mean ± standard deviation. (* *p* < 0.05, ** *p* < 0.01 and *** *p* < 0.001 vs. control, SR-1 1 μM treatment).

To study the effect of SR-1 on the functional ability of senescent EPCs, migration and tube-forming capacity, which are essential for angiogenesis in endothelial cells [38], were assessed. In the scratch-wound healing and transwell migration assays, SR-1 significantly increased the number of migrated cells (Figure 4A,B) and the percentage of the area containing the migrating cells (Figure 4C,D). The tube-formation assay revealed that SR-1 significantly increased the total tube length in the EPCs (Figure 4E,F). We further analyzed the mRNA expression of proangiogenic factors in senescent EPCs. Angiopoietin-1 (Ang1), basic fibroblast growth factor (bFGF) and Interleukin-8 (IL-8) mRNA levels were significantly increased in the SR-1-treated EPCs compared to those in the controls (Figure 4G).

### 3.6. Reduction of AhR Pathway-Mediated Cellular ROS in SR-1-Treated EPCs

**Figure 5:**
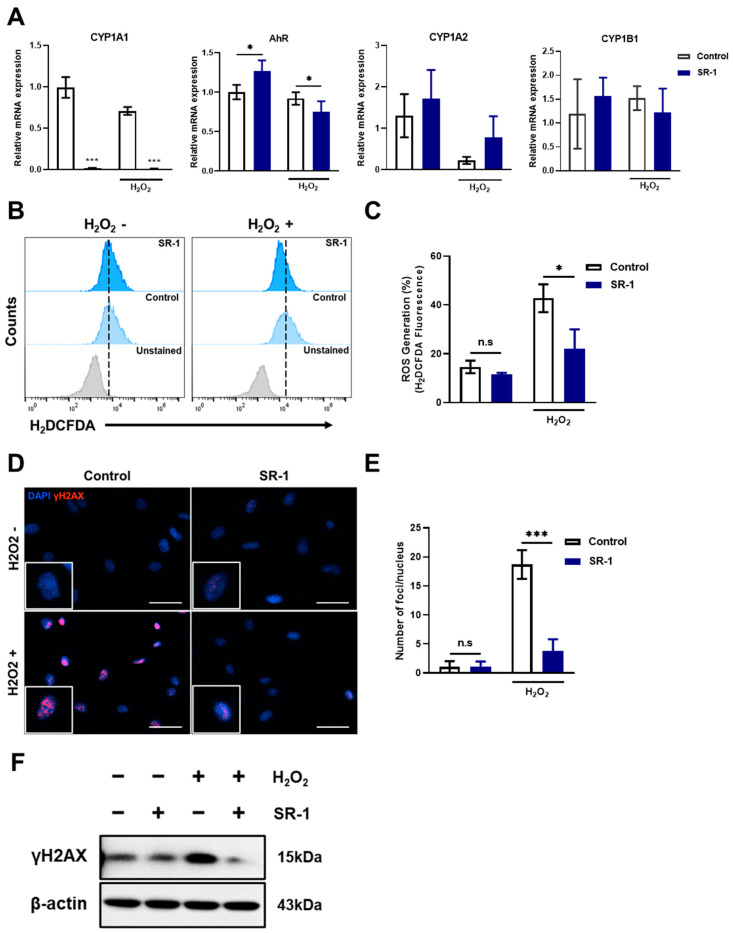
Preservation of SR-1 against H_2_O_2_-induced oxidative stress in EPCs. (**A**) mRNA levels of AhR, CYP1A1, CYP1A2, and CYP1B1 were examined using qRT-PCR. The mRNA levels were normalized to GAPDH. (**B**) ROS production measured using flow cytometry analysis using H_2_DCFDA dye. (**C**) Percentages in the representative histograms indicated the percentage of ROS levels. (**D**) Representative images of γH2A.X (Red) and DAPI (Blue) staining in EPCs (scale bar, 40 μm). The boxed regions are shown at a high magnification (×2) in the inset. (**E**) γH2A.X foci number per nucleus. (**F**) The protein expression of γH2A.X were analyzed via Western blotting. The data are presented as mean ± standard deviation. (n.s represents no significance, * *p* < 0.05 and *** *p* < 0.001 vs. control, SR-1 1 μM treatment, H_2_O_2_ 800 μM treatment for 30 min).

According to previous studies, AhR signaling regulates the expression of numerous CYP enzymes, including members of the cytochrome P450 family 1 (CYP1) [39], which results in ROS accumulation and oxidative stress due to an imbalance between the oxidative and antioxidant systems in the cells [40,41]. Moreover, SR-1 blocked CYP1 expression by direct binding and inhibition of the AhR in previous report [19]. Hence, we hypothesized that in SR-1-treated EPCs, cell maintenance and senescence attenuation are regulated by decreased ROS levels owing to the suppression of CYP1 genes against oxidative stress. We investigated the ROS levels under oxidative stress conditions using H_2_O_2_. First, we examined the suppression of CYP1 mRNA levels under both normal and oxidative stress conditions in SR-1-treated EPCs. CYP1A1, CYP1A2, and CYP1B1, including the ligand AhR, were checked, and CYP1A1 dramatically decreased in the SR-1-treated EPCs, but the difference was not significant in other genes compared to the controls (Figure 5A). This suggests that inhibition of CYP1, especially CYP1A1, was confirmed in SR-1-treated EPCs. H_2_DCFDA showed that preconditioning with SR-1 significantly decreased cellular ROS levels (Figure 5B,C). ROS are known to mediate cellular DNA damage. Additionally, DNA damage is associated with cellular senescence [42,43]. We further assessed the DNA damage marker γH2A.X foci and protein levels. We found that SR-1-treated EPCs prevented DNA damage due to oxidative stress (Figure 5D–F). These results suggest that SR-1 not only reduces ROS production via CYP1A1 suppression but also prevents DNA damage under oxidative stress conditions.

### 3.7. Reduction of CYP1A1-ROS Accumulation-Mediated Cellular Senescence in SR-1-Treated EPCs

**Figure 6:**
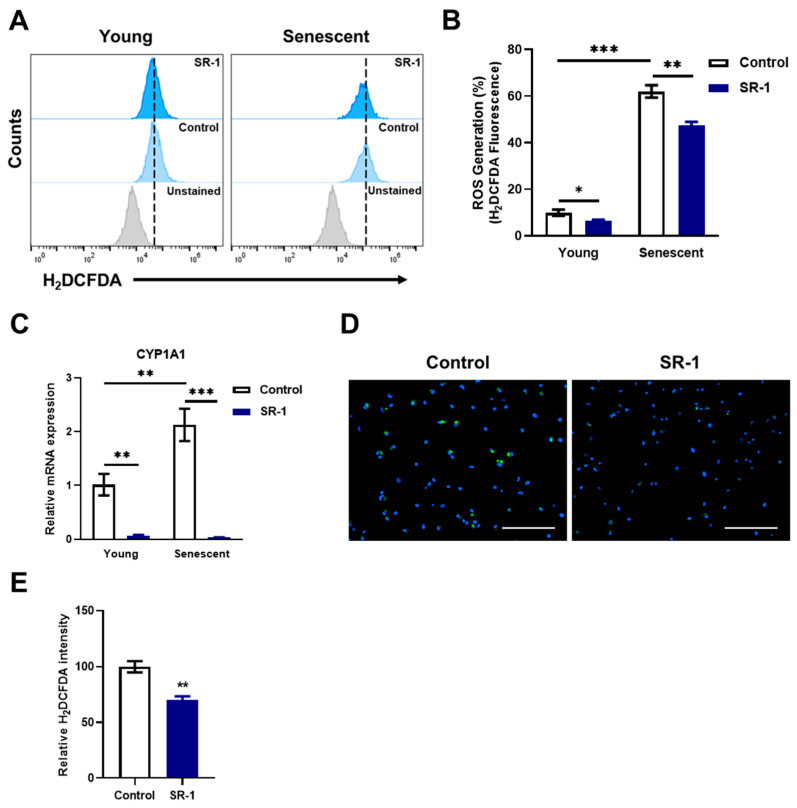
Effect of SR-1 on replicative senescence through ROS accumulation. (**A**) Cellular ROS measured using flow cytometry analysis using H_2_DCFDA dye in young and senescent EPCs. (**B**) Percentages in the representative histograms indicate the percentage of accumulated ROS levels. (**C**) mRNA expression of accumulated CYP1A1 was examined by qRT-PCR. The mRNA levels were normalized to GAPDH. (**D**) Representative H_2_DCFDA (Green) fluorescence images in EPCs with continuous SR-1 treatment. The nucleus was stained by Hoechst-33342 (Blue) (Scale bar, 200 μm). (**E**) The quantified data of H_2_DCFDA fluorescence intensity. The data are presented as mean ± standard deviation. (* *p* < 0.05, ** *p* < 0.01 and *** *p* < 0.001 vs. control, SR-1 1 μM treatment).

The preventive effects of SR-1, such as reducing ROS levels and blocking the AhR pathway, are related to the inhibition of cellular senescence [16,44]. Furthermore, β-galactosidase activity and senescence-associated genes were decreased (Figure 3). To determine whether SR-1 attenuates senescence by inhibiting CYP1A1-ROS accumulation, we investigated the ROS levels and CYP1A1 expression. Intracellular ROS accumulated in senescent EPCs compared to young EPCs and significantly decreased ROS level in the SR-1-treated senescent EPCs compared to senescent EPCs control (Figure 6A,B). CYP1A1 mRNA expression showed a similar pattern to intracellular ROS levels, in which CYP1A1 accumulated in the senescent group and was inhibited in SR-1-treated EPCs (Figure 6C). Finally, it was verified through H_2_DCFDA staining whether the maintenance effect of SR-1 resulted from CYP1A1-ROS reduction. Quantification of live cell image data revealed that intracellular ROS was dramatically reduced in EPCs treated with SR-1 long-term compared to the controls (Figure 6D,E). These data suggest that SR-1 attenuates replicative senescence and preserves its maintenance by inhibiting CYP1A1-mediated ROS accumulation in the EPCs.

### 3.8. Activation of AhR-CYP1A1-Mediated Senescence Acceleration in EPCs

To clarify the protective effect of SR-1 on AhR-CYP1A1 regulation in EPCs senescence, the AhR pathway was activated using an AhR agonist 6-Formylindolo 3 2-b carbazole (FICZ) (Figures S4 and S5) [45]. CYP1A1 mRNA levels were significantly increased in the AhR agonist-treated EPCs compared to controls (Figure S6). To investigate whether activating AhR-CYP1A1 can reverse the attenuation of SR-1 on EPC senescence, we first checked the ROS levels. Intracellular ROS increased more dramatically in the agonist-treated senescent EPCs compared to the controls (Figure S7). Next, we additionally checked the AhR-CYP1A1-mediated EPC phenotypes via long-term treatment of FICZ or SR-1. The morphology of long-term treated EPCs was smaller in SR-1 and larger in the cell area in FICZ (Figure S8). The SA-β-gal activity also reversed the phenotype between the SR-1 and FICZ-treated EPCs compared to control (Figure S9). Moreover, it was confirmed that intracellular ROS of live cell images also decreased in SR-1 and increased in FICZ long-term treated EPCs (Figure S10). These results indicated that the effect of senescence attenuation via SR-1 is due to AhR-CYP1A1 regulation.

### 3.9. Angiogenesis of SR-1-Treated EPCs In Vivo

**Figure 7:**
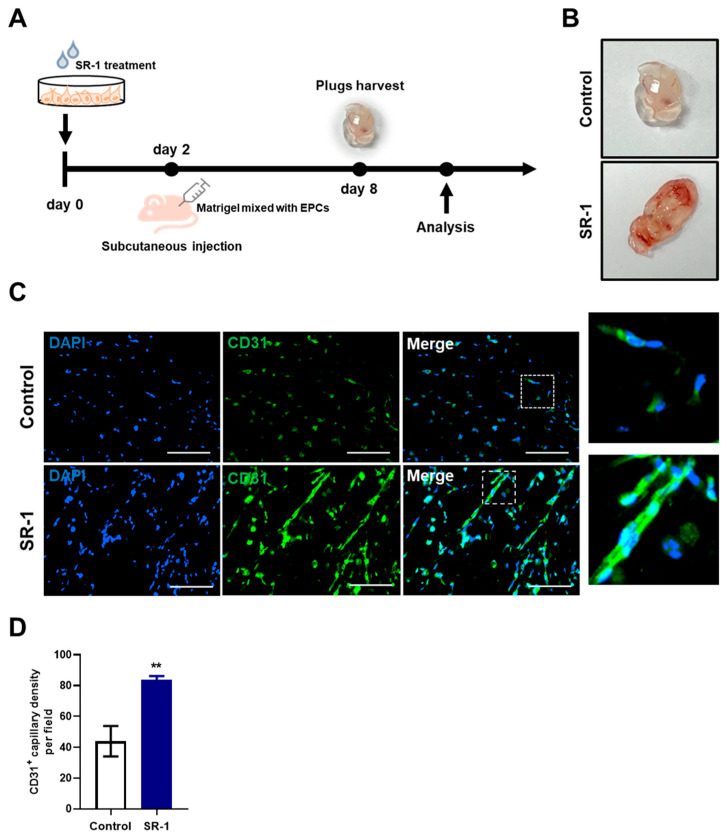
Effect of SR-1 on EPC-mediated angiogenesis. (**A**) Schematic diagram of an in vivo Matrigel plug assay. Matrigel-containing SR-1-treated EPC were subcutaneously injected into nude mice. (**B**) After 6 days, the plugs were collected and photographed. (**C**) For quantitative analysis of angiogenesis, endothelial cells were stained with CD31 (Green). The nucleus was stained by DAPI (Blue) (scale bar, 100 μm). The boxed regions are shown at a high magnification (×4) in the inset. (**D**) percentage of CD31+ capillary density was quantified. The data are presented as mean ± standard deviation. (** *p* < 0.01 vs. control, SR-1 1 μM treatment).

Regulating oxidative stress is associated with cellular damage and ROS, which is important for cell-based therapy [46]. We performed a Matrigel-plug assay in mice to determine whether the protective effect of SR-1 contribute to angiogenesis (Figure 7A). SR-1-treated EPCs became highly vascularized (Figure 7B). Matrigel plugs were further stained for CD31 as marker for endothelial cells. Additional quantification of CD31+ capillary density suggested that SR-1 significantly increased vessel CD31+ vessel-like structures compared to the controls (Figure 7C,D). These results demonstrated that SR-1 protects EPC from stress conditions and improves angiogenesis in vivo as well as ex vivo.

## 4. Discussion

This study aimed to investigate the effect of SR-1 on EPCs maintenance. This was the first study to report the effect of SR-1 in EPCs and describe the attenuation of EPC senescence by inhibiting AhR signaling pathway. We demonstrated that SR-1 contributes to maintaining EPC characteristics, including functional ability against replicative cellular senescence. Moreover, we also found that SR-1, which blocks AhR-mediated CYP1A1 production, protects EPC from oxidative stress including ROS in vitro, and this effect contributes to angiogenic potential also in vivo.

We demonstrated the long-term maintenance of CD34+ populations in SR-1 long-term-treated EPCs (Figure 1B). These cells also preserved their EPC characteristics, including cell morphology, cell yield and proliferative capacity (Figure 2). SR-1 is an AhR antagonist that has been shown to expand CD34+ HSC populations [19]. Several CD34+ population studies using SR-1 have also been published on the expansion effect [21,22,23]. In addition, clinical trials using SR-1-expanded hematopoietic stem cells have shown improved patient recovery [20]. CD34 is a cell-surface glycoprotein that functions as a cell-to-cell adhesion molecule expressed on various stem and progenitor cells, including vascular endothelial progenitor cells [36]. It has been reported that CD34+ cells induce therapeutic angiogenesis in several ischemic diseases, including myocardial ischemia, ischemic stroke, and ischemic hindlimb [47,48,49]. In contrast, CD34− cells contribute to the improvement of blood vessel formation and preservation of progenitor status with CD34+ cells as niche-supporting cells [50]. Coexistence of the two cells is important because interactions with CD34− cells are also involved in EPC maintenance. Our study supports previous studies on the effect of SR-1 on CD34 population and the importance of CD34 in EPCs and proposes the efficiency of using SR-1 in EPC studies.

Maintaining the characteristics of stem cells is closely related to aging [51]. We investigated the senescent phenotype of the long term cultured or aged EPCs. It confirmed the effect of SR-1 through the reduction of SA-β-gal and SASP, which are representative phenotypes of aging in senescent EPCs (Figure 3A,B,E). In addition, DNA damage, ROS, and cell cycle inhibitors such as p53 and p21 are upregulated in senescent cells. [51]. Moreover, SASP and ROS can induce senescence through a positive feedback loop [52]. We confirmed that SR-1 reduces the amount of intracellular ROS and the expressions of p53/p21 in senescent EPCs (Figure 3C and Figure 6A,B). We also found that the expression of γH2A.X, which serves as a marker of DNA double-strand break, was decreased along with ROS under oxidative stress conditions (Figure 5D–F). These data suggested that SR-1 maintains EPC by reducing the risk factors of aging. Functional ability was conducted in SR-1-treated senescent EPCs (Figure 4). Cellular aging results in impairment of cellular function and regeneration [53]. Endothelial cell senescence leads to a decrease in essential functions, such as proliferation, migration, and differentiation for stem cell-based research and therapy [54]. We demonstrated that SR-1 contributes to the protection of functional capacity on cellular aging in EPCs.

We demonstrated the ROS reduction phenotype in SR-1-treated EPCs by antagonizing AhR-CYP1A1 activation in H_2_O_2_-induced oxidative stress (Figure 5). AhR is a ligand-dependent transcription factor that regulates gene expression [24]. It is crucial for various physiological processes and functions in several cell types [55]. AhR activation leads to the transcriptional activation of CYP1A1, CYP1A2, and CYP1B1, which are enzymes involved in oxidative activation. Moreover, in various research cases, it has been reported that CYP1A1 was predominantly highly expressed in mRNA level and enzyme activity [56]. These studies support our reason for why only significant changes in CYP1A1 expression were observed under our experimental conditions in SR-1-treated EPCs. In addition, in our experimental data, there was little difference in SR-1 treated EPC under normal conditions (not aging or stress-induced conditions) compared to control. However, oxidative stress-induced and replicative cellular senescence conditions intensified CYP1A1 and ROS generation, and the blocking effects of the AhR-CYP1A1-ROS pathway were more dramatic than normal conditions in SR-1-treated EPCs (Figure 6A–C). AhR and CYP genes are closely related in oxidative stress and aging process [44,57]. The difference in these results may be due to excessive expression of CYP genes by accelerated ROS production in cells under aging or oxidative stress conditions. AhR can activate several signaling pathways that regulate multiple cellular functions [58]. Furthermore, AhR activation is associated with oxidative stress and antioxidative response, such as Nrf2 activation [32]. Interestingly, we confirmed the increased AhR pattern in the SR-1 treated cells, which differed from CYP1A1 expression (Figure 5A). Previous studies have suggested that Nrf2 binds to the AhR promotor and increases the mRNA transcription of AhR [59]. These findings may have influenced the increased AhR results. These suggest that further studies are needed to confirm additional mechanisms involved in the effects of SR-1.

Following the assessment of functionality in vitro, we also verified the effect in vivo. In cell-based therapy, in vivo transplantation efficiency and ex-vivo cell yield and function are important [60]. SR-1 treated EPCs were demonstrated in vivo through enhanced angiogenic potential in Matrigel plugs (Figure 7). This suggests that SR-1 showed a protective effect during angiogenesis in EPCs against cellular stress conditions in vivo. Additionally, we showed slight effects of SR-1 under normal conditions at a concentration that did not affect EPC viability. SR-1 distinctly maintained EPCs during oxidative stress and replicative cellular senescence. This suggests that EPCs do not disrupt normal cell maintenance via SR-1 and only protect against stress sources during ex vivo expansion. Furthermore, the efficacy of SR-1 has been proven in clinical trials using hematopoietic stem cells [20]. These results can also be used to research supplements in cell culture media and drug delivery applications [61,62,63]. It has been proposed that SR-1 is a suitable factor for EPC and blood vessel regeneration studies.

## 5. Conclusions

These findings suggest that stimulation of EPCs with SR-1 contributes to the maintenance of EPCs, including CD34+ progenitor populations, cells characteristics, and the attenuation of senescence. It was revealed that the SR-1 effects were conducted through antagonization of AhR-CYP1A1-mediated ROS generation. As a result, SR-1 blocks the binding between AhR and the ligand, reducing excessive production of CYP1A1 and ROS due to aging during in vitro long-term culture. Furthermore, these effects improved the functional ability of EPCs both in vitro and in vivo. These results demonstrate the potential of SR-1 as an efficient therapeutic agent for EPC-based therapies in vascular diseases.

## Data Availability

The data used to support the findings of this study are included in the article and within the supplementary file. Further inquiries can be directed to the corresponding author.

## Supplementary Materials

The following supporting information can be downloaded at: https://www.mdpi.com/article/10.3390/cells12152005/s1.
