# Supplementary material for: StemRegenin-1 Attenuates Endothelial Progenitor Cell Senescence by Regulating the AhR Pathway-Mediated CYP1A1 and ROS Generation
Source: Cells. 2023 Aug 5;12(15):2005. doi: 10.3390/cells12152005 (PMC10417434; doi:10.3390/cells12152005)
Supplement: Supplementary file 1 [file cells-12-02005-s001.zip › cells-2518692-supplementary.pdf]

cells

MDPI

Supplementary Figures and Table

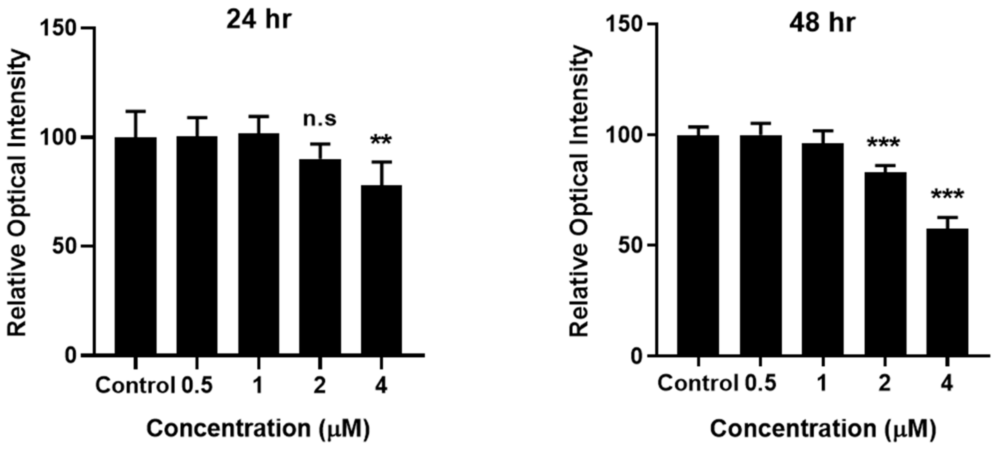

Figure S1. Cell viability of EPCs treated with various concentrations of SR-1 for 24 h and 48 h as measured by CCK cell viability assay. The data are presented as mean ± standard deviation. (**p<0.01 and ***p<0.001 vs. control)

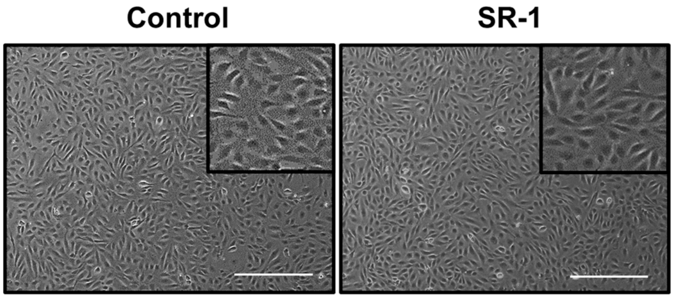

Figure S2. Morphology of SR-1(1 μM)-treated EPCs (scale bar, 400 μm). The boxed regions are shown at a high magnification (×2) in the inset.

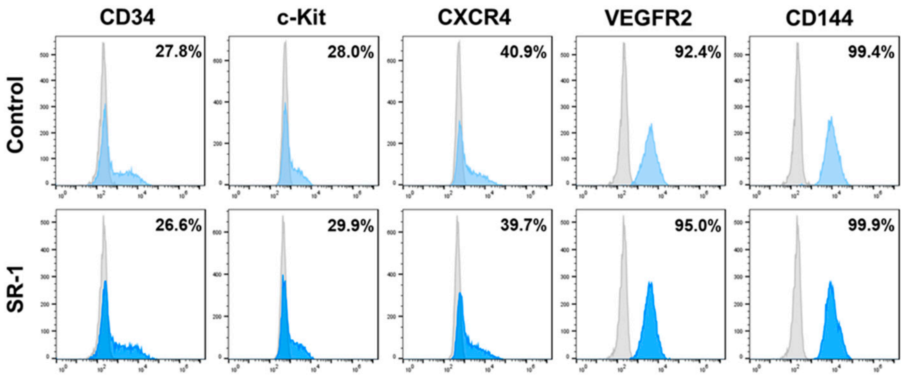

Figure S3. Flow cytometry analysis of cell surface markers of EPC (CD34, c-Kit, CXCR4, VEGFR-2, and CD144) in controls or SR-1-treated EPCs.

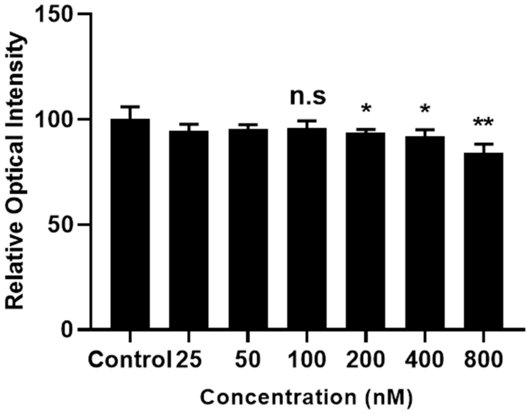

Figure S4. Cell viability of EPCs treated with various concentrations of FICZ for 48 h as measured by CCK cell viability assay. (n.s represents no significance, *p<0.05 and **p<0.01 vs. control)

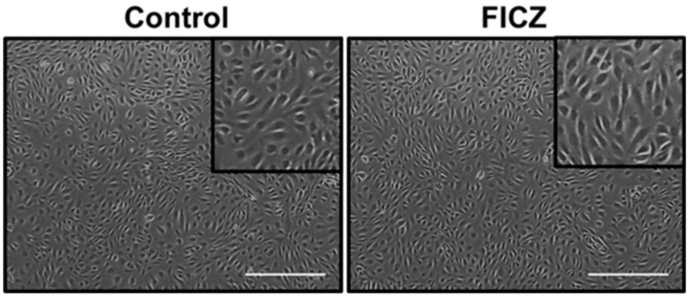

Figure S5. Morphology of FICZ (100 nM)-treated EPCs (scale bar, 400 μm). The boxed regions are shown at a high magnification (×2) in the inset.

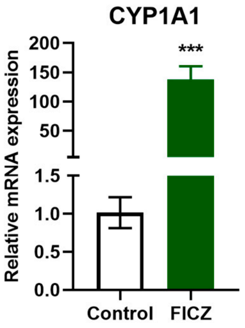

Figure S6. CYP1A1 mRNA levels were examined using qRT-PCR. The mRNA levels were normalized to GAPDH. (***p<0.001 vs. control, FICZ 100 nM treatment)

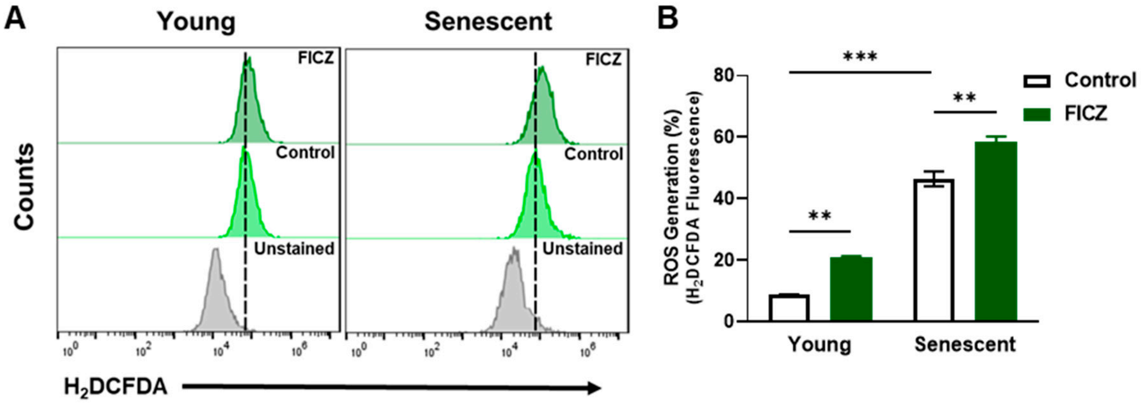

Figure S7. (A) Flow cytometry analysis was used for cellular ROS measurement using H2DCFDA dye in young and senescent EPCs. (B) Percentages in the representative histograms indicate the accumulated ROS levels. (*p<0.05 and ***p<0.001 vs. control, FICZ 100 nM treatment)

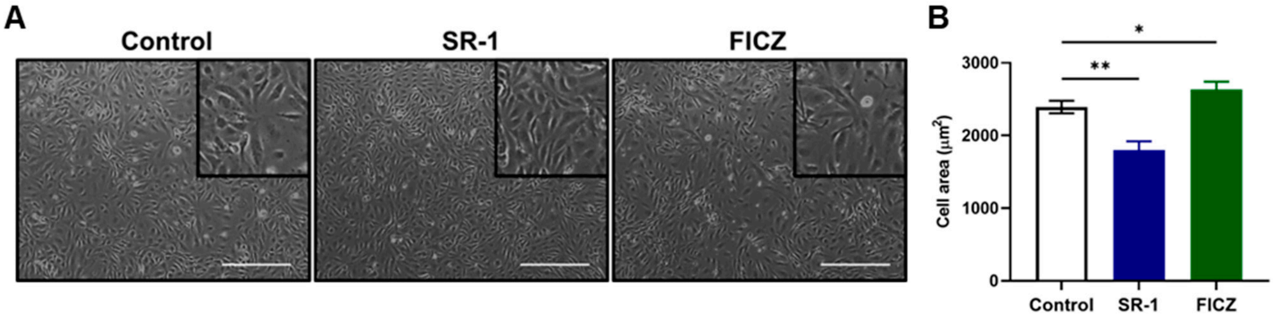

Figure S8. (A) Representative phage-contrast images for long-term culture of EPCs with continuous FICZ or SR-1 treatment (Scale bar, 400 μm). (B) The boxed regions are shown at a high magnification (×2) in the inset. Cell morphometric parameters measuring cell area. (*p<0.05 and **p<0.01 vs. control, FICZ 100 nM and SR-1 1 μM treatment)

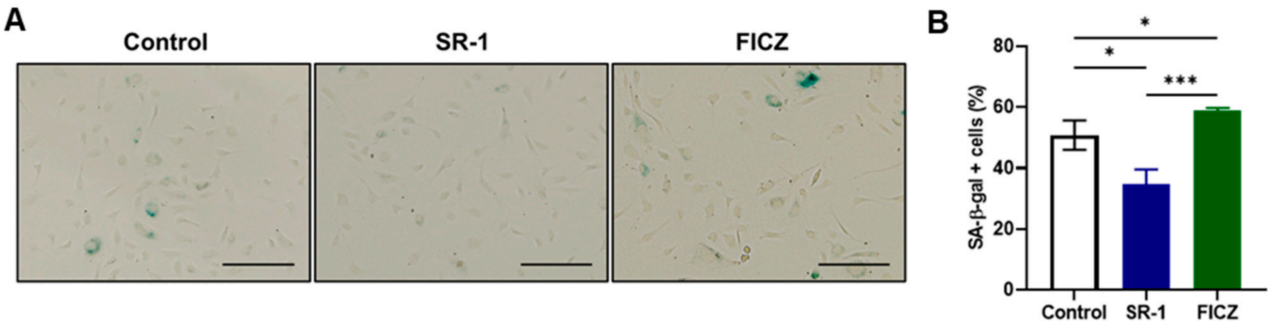

Figure S9. (A) SA-β-gal activity was measured in FICZ or SR-1-continuously treated EPCs (Scale bar, 200 μm). (B) Percentage of SA-β-gal+ cells. (*p<0.05 and ***p<0.001 vs. control, FICZ 100 nM and SR-1 1 μM treatment)

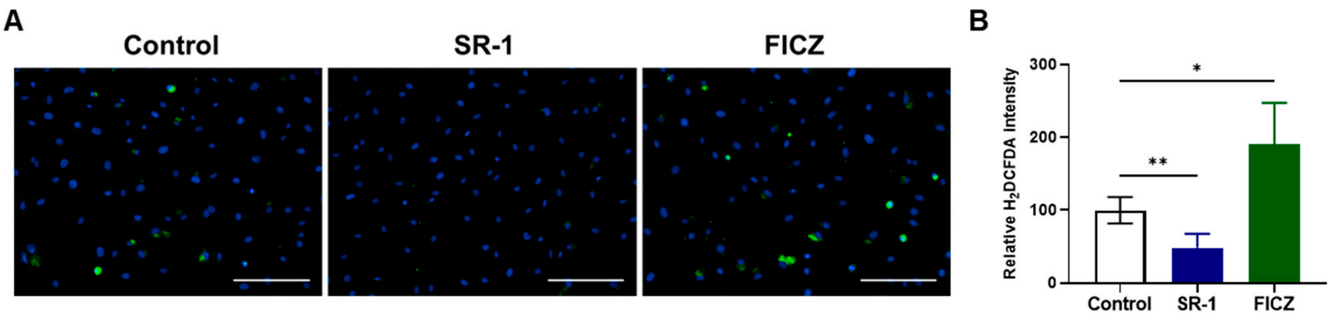

Figure S10. (A) H2DCFDA (green) fluorescence representative images in EPCs with continuous FICZ or SR-1 treatment. The nucleus was stained by Hoechst-33342 (Blue) (Scale bar, 200 μm). (B) The quantified data of H2DCFDA fluorescence intensity. The data are presented as mean ± standard deviation. (*p<0.05 and **p<0.01 vs. control, FICZ 100 nM and SR-1 1 μM treatment)

Table S1. Primer sequence for quantitative RT-PCR

Gene	Primer	sequences
AhR	Forward	5′- *CTTCCAAGCGGCATAGAGAC* -3′
	Reverse	5′- *AGTTATCCTGGCCTCCGTTT* -3′
CYP1A1	Forward	5′- CTTGGACCTCTTTGGAGCTG -3′
	Reverse	5′- CGAAGGAAGAGTGTCGGAAG -3′
CYP1A2	Forward	5′- CAATCAGGTGGTGGTGTCAG -3′
	Reverse	5′- GCTCCTGGACTGTTTTCTGC -3′
CYP1B1	Forward	5′- TGTCCTGGCCTTCCTTTATG -3′
	Reverse	5′- TCATCACTCTGCTGGTCAGG -3′
IL-6	Forward	5′- TACCCCCAGGAGAAGATTCC -3′
	Reverse	5′- TTTTCTGCCAGTGCCTCTTT -3′
IL-1α	Forward	5′- AATGACGCCCTCAATCAAAG -3′
	Reverse	5′- TGGGTATCTCAGGCATCTCC -3′
Ang1	Forward	5′- AAATGGAAGGAAAACACAAGGAA -3′
	Reverse	5′- ATCTGCACAGTCTCTAAATGGT -3′
bFGF	Forward	5′- TTCCTTCATAGCCAGGTAACGG -3′
	Reverse	5′- ATCTGCACAGTCTCTAAATGGT -3′
IL-8	Forward	5′- GTGCAGTTTTGCCAAGGAGT -3′

	Reverse	5′- CTCTGCACCCAGTTTTCCTT -3′
GAPDH	Forward	5′- ACCACAGTCCATGCCATCAC -3′
	Reverse	5′- TCCACCACCCTGTTGCTGTA -3′
